# Effects of Near-Infrared Diode Laser Irradiation on Pain Relief and Neuropeptide Markers During Experimental Tooth Movement in the Periodontal Ligament Tissues of Rats: A Pilot Study

**DOI:** 10.3390/ijms26157404

**Published:** 2025-07-31

**Authors:** Kanako Okazaki, Ayaka Nakatani, Ryo Kunimatsu, Isamu Kado, Shuzo Sakata, Hirotaka Kiridoshi, Kotaro Tanimoto

**Affiliations:** Department of Orthodontics and Craniofacial Development Biology, Graduate School of Biomedical & Health Sciences, Hiroshima University, Hiroshima 734-8553, Japan; okanako@hiroshima-u.ac.jp (K.O.); anakatan@hiroshima-u.ac.jp (A.N.);

**Keywords:** photobiomodulation, near-infrared diode laser, neuropeptides, substance P, calcitonin gene-related peptide, heat shock protein 70, periodontal tissues

## Abstract

Pain following orthodontic treatment is the chief complaint of patients undergoing this form of treatment. Although the use of diode lasers has been suggested for pain reduction, the mechanism of laser-induced analgesic effects remains unclear. Neuropeptides, such as substance P (SP) and calcitonin gene-related peptide (CGRP), contribute to the transmission and maintenance of inflammatory pain. Heat shock protein (HSP) 70 plays a protective role against various stresses, including orthodontic forces. This study aimed to examine the effects of diode laser irradiation on neuropeptides and HSP 70 expression in periodontal tissues induced by experimental tooth movement (ETM). For inducing ETM for 24 h, 50 g of orthodontic force was applied using a nickel–titanium closed-coil spring to the upper left first molar and the incisors of 20 male Sprague Dawley rats (7 weeks old). The right side without ETM treatment was considered the untreated control group. In 10 rats, diode laser irradiation was performed on the buccal and palatal sides of the first molar for 90 s with a total energy of 100.8 J/cm^2^. A near-infrared (NIR) laser with a 808 nm wavelength, 7 W peak power, 560 W average power, and 20 ms pulse width was used for the experiment. We measured the number of facial groomings and vacuous chewing movements (VCMs) in the ETM and ETM + laser groups. Immunohistochemical staining of the periodontal tissue with SP, CGRP, and HSP 70 was performed. The number of facial grooming and VCM periods significantly decreased in the ETM + laser group compared to the ETM group. Moreover, the ETM + laser group demonstrated significant suppression of SP, CGRP, and HSP 70 expression. These results suggest that the diode laser demonstrated analgesic effects on ETM-induced pain by inhibiting SP and CGRP expression, and decreased HSP 70 expression shows alleviation of cell damage. Thus, although further validation is warranted for human applications, an NIR diode laser can be used for reducing pain and neuropeptide markers during orthodontic tooth movement.

## 1. Introduction

Orthodontic tooth movement (OTM) is performed by applying mechanical force to teeth. However, orthodontic force application to the jawbone or teeth causes pain as a side effect. All orthodontic treatment procedures, such as the placement of separators, placement and activation of the arch wire, and debonding, cause pain in patients [1,2,3]. The most common reason for patients not opting for orthodontic treatment is the pain associated with it [4]. Surveys on the percentage of patients experiencing pain have reported values ranging from 70 to 95% [2]. Previous reports have even stated that approximately 30% of patients have considered discontinuing treatment owing to pain [4,5]. Moreover, orthodontic pain decreases the health-related quality of life of patients by interfering with daily activities such as eating and talking [1,4,6].

The mechanism of orthodontic treatment-induced pain involves a non-infectious inflammatory response triggered by mechanical forces on the periodontal tissue and neurogenic inflammation caused by the release of neuropeptides from nociceptive fibers [7,8]. Nociceptive signals generated by damage to the periodontal tissues are relayed to the spinal tract nucleus of the trigeminal nerve and transmitted to the sensory center of the cerebral cortex, where they are perceived as pain [9].

We previously investigated the mechanism of pain relief by diode laser irradiation. When pain is induced by orthodontic force application to the experimental tooth movement (ETM) model in rats, the expression of glial fibrillary acidic protein (GFAP), a marker of chronic pain and astrocytes, is enhanced in the maxillary innervated region of the trigeminal spinal tract angle, which is considered an intermediate pathway in the cerebral cortex. Moreover, we found that GFAP expression was significantly suppressed by diode laser irradiation of the periodontal tissue [10]. Moreover, diode laser irradiation suppressed the expression of prostaglandin (PG) E2, which increases pain, by suppressing the release of cyclooxygenase (COX) 2 and interleukin-1β (IL-1β) from the periodontal ligament cells and gingival fibroblasts [11]. These results strongly suggest that diode laser irradiation has a pain-relieving effect on ETM in rats.

Many studies have examined the histological changes associated with OTM. Other reports have shown that neuropeptides involved in the mechanism of pain caused by tooth movement, such as substance P (SP) and calcitonin gene-related peptide (CGRP), are expressed in the periodontal tissue during experimental tooth movement (ETM) [12,13,14,15]. However, no reports have examined the changes in their expression due to 808 nm diode laser irradiation. Other reports have demonstrated that the expression of heat shock protein (HSP) 70, which plays important roles in maintaining cellular homeostasis to withstand stress, is increased in the periodontal tissue by ETM, and its expression is suppressed by laser irradiation [16,17,18]. However, only one study has reported reduced HSP70 levels by laser irradiation [16], and no study has reported on its association with pain. This study aimed to examine the relationship between the expression of SP, CGRP, and HSP 70 and pain caused by ETM and the effects of diode laser irradiation.

## 2. Results

### 2.1. Effects of Diode Laser Irradiation on Nociceptive Behaviors in ETM

**Figure 1:**
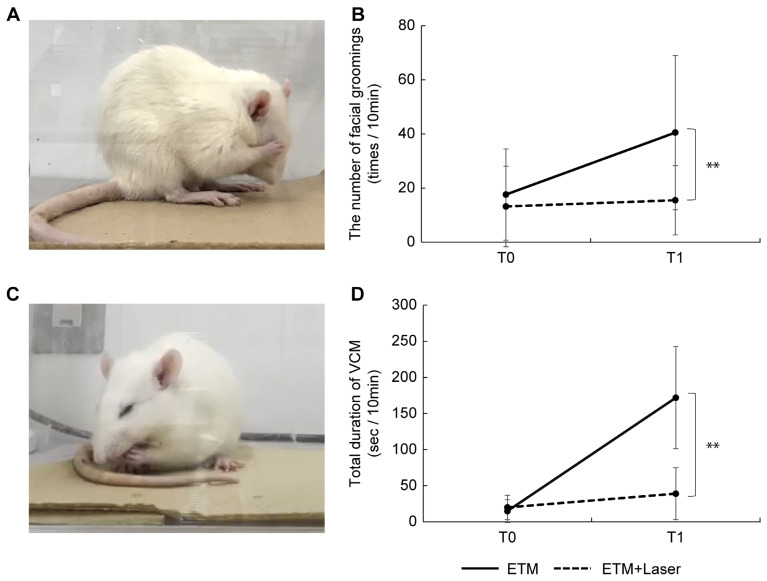
Effects of diode laser irradiation on nociceptor behavior (facial grooming and vacuous chewing movement) during experimental tooth movement. (**A**) Images captured from a video showing the facial grooming of rats in pain. (**B**) The number of facial grooming events in the experimental tooth movement (ETM) + laser group at T1 was significantly lower than that in the ETM group at T1 (*p* < 0.01). At T0, no significant differences were observed between the ETM and ETM + laser groups (*p* = 0.499). (**C**) Images captured from a video showing the vacuous chewing movement of the rats in pain. (**D**) The vacuous chewing movement period in the ETM + laser group at T1 was significantly shorter than that in the ETM group at T1 (*p* < 0.01). At T0, no significant differences were observed between the ETM and ETM + laser groups (*p* = 0.718). *n* = 10. ** *p* < 0.01 (Tukey’s test).

An image of facial grooming captured from a videotape is shown in Figure 1A. In the ETM group, the number of facial groomings was significantly increased at T1 compared with T0 (17.6 ± 16.9 vs. 40.5 ± 28.5, *p* < 0.05). In the ETM + laser group, no significant differences were observed in the number of facial groomings between T0 and T1 (13.2 ± 14.9 vs. 15.5 ± 12.8, *p* = 0.260). At T0, no significant differences were observed between the ETM group and ETM + laser group (17.6 ± 16.9 vs. 13.2 ± 14.9, *p* = 0.499). A significant reduction in the number of facial groomings was observed in the ETM + laser group compared to the ETM group at T1 (40.5 ± 28.5 vs. 15.5 ± 12.8, *p* < 0.01, Figure 1B).

An image of the vacuous chewing movements (VCMs) captured from the video is shown in Figure 1C. A significant increase was observed in the VCM period in the ETM group at T1 compared to the ETM group at T0 (14.9 ± 15.9 vs. 172.0 ± 70.6, *p* < 0.01). In the ETM + laser group, the VCM period tended to increase more at T1 than at T0; however, the differences were not significant (19.9 ± 16.9 vs. 39.0 ± 36.0, *p* = 0.139). Moreover, no significant differences were observed between the VCM period in the ETM group and the ETM + laser group at T0 (14.9 ± 15.9 vs. 19.9 ± 16.9, *p* = 0.718). A more significant reduction was observed in the VCM period in the ETM + laser group than in the ETM group at T1 (172.0 ± 70.6 vs. 39.0 ± 36.0, *p* < 0.01, Figure 1D).

### 2.2. Effects of Diode Laser Irradiation on Temperature

**Figure 2:**
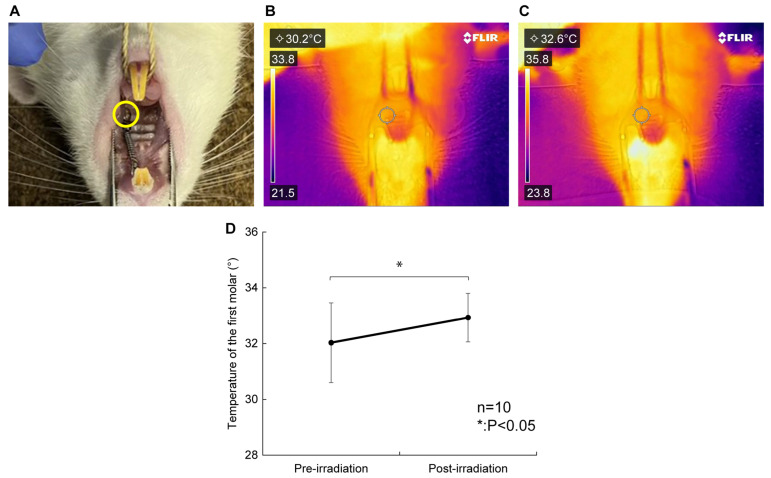
Effects of diode laser irradiation on temperature. (**A**) The temperature of the upper left first molar of the rat, the irradiated site (yellow circle), was measured. (**B**) FLIR camera thermogram before irradiation. The measured temperatures are shown in the upper left. (**C**) FLIR camera thermogram after irradiation. The measured temperatures are shown in the upper left. (**D**) The temperature of the periodontal tissue increased significantly post-irradiation compared with pre-irradiation (32.0 ± 1.4 vs. 32.9 ± 0.9 °C, *p* < 0.05). The average temperature change was +0.9 °C, and no increase of more than 2.4 °C was observed. *n* = 10. * *p* < 0.05, compared to pre-irradiation (Wilcoxon signed-rank test).

The surface temperature of the periodontal tissue was determined through thermography before (Figure 2B) and after irradiation (Figure 2C). The temperature around the rat’s maxillary left first molar, which was the irradiated site, increased significantly after laser irradiation compared to that before laser irradiation (32.0 ± 1.4 vs. 32.9 ± 0.9 °C, *p* < 0.05, Figure 2D). The average temperature change before and after laser irradiation was +0.9 °C, and no increase of more than 2.4 °C was observed. The maximum temperature measured after laser irradiation was 34.0 °C.

### 2.3. Immunohistochemical Staining

#### 2.3.1. Substance P Immunohistochemical Staining

**Figure 3:**
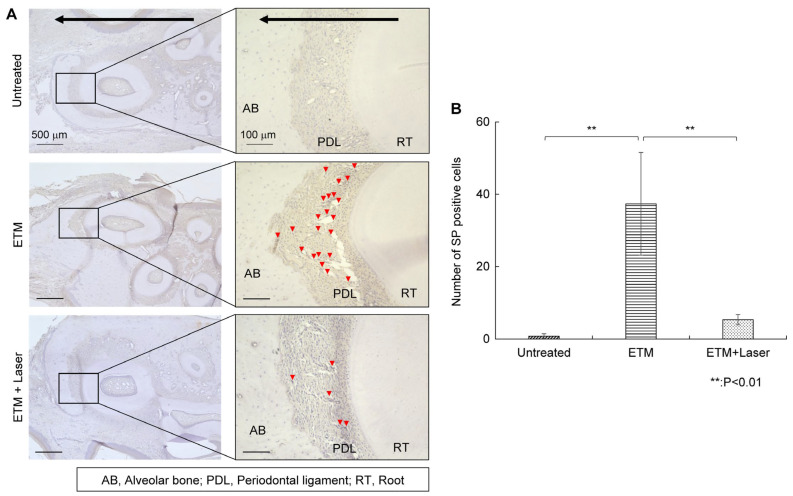
Immunohistochemical staining for substance P. Expression of substance P in the periodontal tissues. (**A**) As tooth movement occurs in the direction of the arrow (black) in the figure, the left side of each root was considered the compression side. The arrowheads indicate P-positive cells (red). (**B**) The number of substance P-positive cells in the periodontal ligament on the compression side in the untreated, ETM, and ETM + laser groups. *n* = 5. ** *p* < 0.01 (Tukey’s tests). Scale bar, 500 μm in the left-hand slides (×4); 100 μm in the right-hand slides (×20). AB, alveolar bone; PDL, periodontal ligament; RT, root.

Almost no SP expression was observed on the compressed side of the periodontal ligament in rats in the untreated group (Figure 3A). The ETM group demonstrated increased expression of SP in the periodontal ligament compared to the untreated group (Figure 3A). Moreover, ETM-enhanced SP expression was suppressed by diode laser irradiation (Figure 3A). The SP-positive cells observed through immunohistochemical (IHC) staining are shown in Figure 3A, and the number of SP-positive cells in each group is shown in Figure 3B. The expression level of SP in the periodontal ligament increased significantly (37.4 ± 15.9, *p* < 0.01) in the ETM group compared to the untreated group (0.8 ± 0.7). Rats in the ETM + laser group exhibited significantly fewer SP-positive cells than those in the ETM group (5.3 ± 1.6 vs. 37.4 ± 15.9 cells, *p* < 0.01). Although no significant difference was observed in the number of SP-positive cells between the untreated and ETM + laser groups, the ETM + laser group had a higher number of SP-positive cells than the untreated group (5.3 ± 1.6 vs. 0.8 ± 0.7 cells, *p* = 0.725).

#### 2.3.2. CGRP IHC Staining

**Figure 4:**
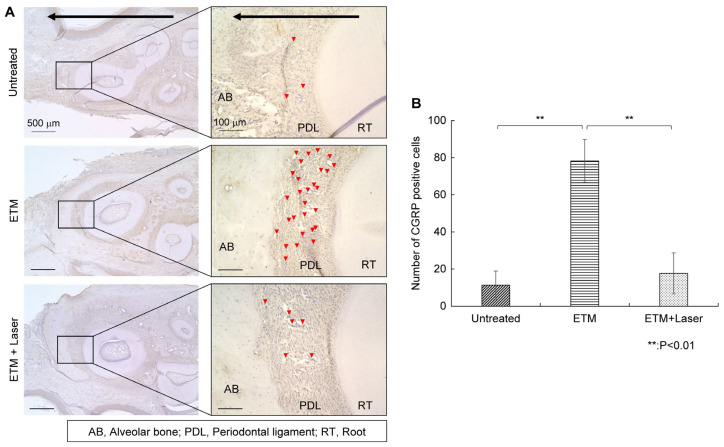
Immunohistochemical staining. CGRP expression in the periodontal tissues. (**A**) As tooth movement occurs in the direction of the arrow (black) in the figure, the left side of each root was considered the compression side. The arrowheads indicate CGRP-positive cells (red). (**B**) The number of CGRP-positive cells in the periodontal ligament on the compression side in the untreated, ETM, and ETM + laser groups. *n* = 5. ** *p* < 0.01 (Tukey’s tests). Scale bar, 500 μm in the left-hand slides (×4); 100 μm in the right-hand slides (×20). CGRP, calcitonin gene-related peptide; AB, alveolar bone; PDL, periodontal ligament; RT, root.

Rats in the ETM group exhibited increased CGRP expression in the periodontal ligament compared to those in the untreated group (Figure 4A). The enhanced CGRP expression induced by ETM was suppressed by diode laser irradiation (Figure 4A). CGRP-positive cells observed through IHC staining are shown in Figure 4A, and the number of CGRP-positive cells on the compression side is shown in Figure 4B. Rats in the ETM group had a significantly increased number of CGRP-positive cells compared to those in the untreated group (78.3 ± 13.0 vs. 11.3 ± 8.5 cells, *p* < 0.01). Moreover, the number of CGRP-positive cells increased as a result of ETM was significantly decreased by diode laser irradiation (17.6 ± 12.4 vs. 78.3 ± 13.0 cells, *p* < 0.01). No significant differences were observed in the number of CGRP-positive cells between the untreated and ETM + laser groups (17.6 ± 12.4 vs. 11.3 ± 8.5 cells, *p* = 0.666).

#### 2.3.3. HSP 70 IHC Staining

**Figure 5:**
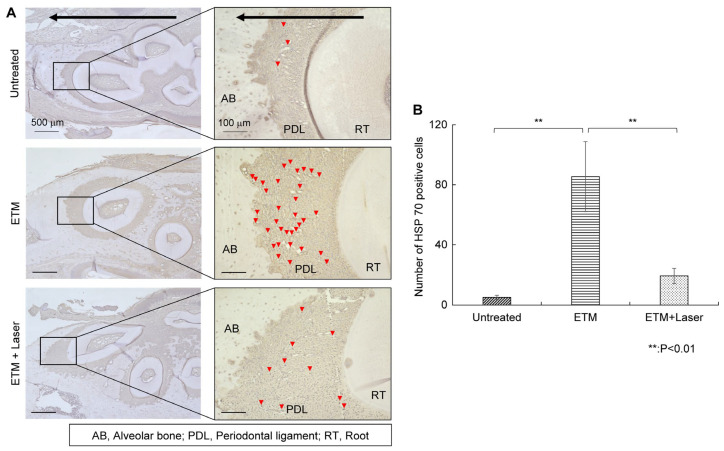
HSP 70 immunohistochemical staining. The expression of HSP 70 in the periodontal tissues. (**A**) As tooth movement occurs in the direction of the arrow (black) in the figure, the left side of each root was considered the compression side. Arrowheads indicate HSP 70-positive cells (red). (**B**) Number of HSP 70-positive cells in the periodontal ligament on the compression side in the untreated, ETM, and ETM + laser groups. *n* = 5. ** *p* < 0.01 (Tukey’s tests). Scale bar, 500 μm in the left-hand slides (×4); 100 μm in the right-hand slides (×20). HSP 70, heat shock protein 70; AB, alveolar bone; PDL, periodontal ligament; RT, root.

Rats in the ETM group demonstrated increased expression of HSP 70 in the periodontal ligament on the compression side compared to those in the untreated group (Figure 5A). HSP 70 expression enhanced by ETM was suppressed by diode laser irradiation (Figure 5A). The number of HSP 70-positive cells in each group is shown in Figure 5B. The number of HSP 70-positive cells was significantly greater in the ETM group than in the untreated group (85.5 ± 26.1 vs. 5.0 ± 1.5 cells, *p* < 0.01). Moreover, the number of HSP 70-positive cells increased by ETM was significantly decreased by diode laser irradiation (19.3 ± 5.7 vs. 85.5 ± 26.1 cells, *p* < 0.01). Although compared to the untreated group, the expression level of HSP 70 in the periodontal ligament increased in the ETM + laser group, no significant differences were observed in the number of HSP 70-positive cells between the untreated and ETM + laser groups (5.0 ± 1.5 vs. 19.3 ± 5.7 cells, *p* = 0.344).

## 3. Discussion

Both facial grooming behavior and VCMs have frequently been used to evaluate nociception in ETM models [10,19,20,21,22,23,24,25]. Several in vivo studies using the ETM model have reported that the maximum nociceptive behavior induced by ETM is attained on day 1 [20,21,22,23,25]. Therefore, in this study, we evaluated nociceptor behavior in rats 24 h after ETM. We observed that the number of facial grooming and VCM periods significantly increased with ETM (Figure 1). Furthermore, diode laser irradiation significantly decreased the number of facial grooming behaviors and the VCM period. Therefore, diode laser irradiation seemingly suppressed the pain induced by ETM.

A previous study revealed that diode lasers suppress GFAP expression in the central nervous system [10]. Hence, we examined their effects on periodontal tissues. As a mechanism of pain generated by orthodontic forces in periodontal tissue, when orthodontic force is applied to the teeth, blood vessels on the compression side are squeezed, resulting in local ischemia [26]. Local ischemia leads to increased anaerobic respiration in the periodontal cells, and the local proton concentration increases. Local acidic conditions stimulate the opening of acid-sensing ion channel 3 (ASIC3) and transient receptor potential vanilloid 1 (TRPV1) channels, subsequently promoting the release of SP and CGRP both centrally (trigeminal nucleus) and peripherally (periodontal tissues), strengthening signal transmission. These neurogenic mediators cause local vasodilation and increase local inflammation and pain sensation.

CGRP is synthesized in the trigeminal ganglion (TG) neurons and released into peripheral periodontal tissues [12], which transmit the signal to the TG to increase the excitability of the trigeminal nerve [1]. Moreover, CGRP has an effect on satellite glial cells (SGCs), upregulating the expression of nitric oxide in the p38 signaling pathway, thereby accelerating the release of signal molecules that stimulate neurons, promoting pain [27]. Moreover, CGRP promoter activity is stimulated in response to tumor necrosis factor-α (TNFα) activation of mitogen-activated protein kinase (MAPK). In this study, we confirmed that ETM increased the expression of CGRP in the periodontal ligament on the compression side and that this expression was suppressed by diode laser irradiation (Figure 4). Laser irradiating the periodontal tissue presumably suppresses pain generation. However, it was not clear whether this was suppressed inflammation-induced CGRP or the CGRP released from peripheral nerves in the present experiment. ASIC3 expression reportedly increases in the periodontal tissues [28], and TRPV1 expression increases in the TG and periodontal tissues 1, 3, and 5 days after ETM [23,24,29,30]. To better understand the mechanism of pain relief following laser irradiation, it is necessary to examine the relationship between the expression of these channels and laser irradiation.

SP is a neuropeptide produced in a subset of capsaicin-sensitive sensory peripheral neuronal cell bodies localized in the dorsal root and TG [13,31]. SP causes immune cells to release inflammatory mediators and activates nociceptors [32]. SP can promote IL-1β and TNF-α through the neurokinin-1 (NK-1) receptor and upregulate the phosphorylation of the MAPK, extracellular signal-regulated kinase (ERK), and p38 pathways to activate SGCs and regulate the inflammatory process [33]. SP can also increase the production of PGE2 and COX2 expression through the NK-1 receptor [33] A previous study demonstrated that IL-1β, PGE2, and COX2 in periodontal tissues following ETM were suppressed by laser irradiation [11]. In this study, SP expression, which was increased by ETM, was decreased by diode laser irradiation (Figure 3). Therefore, the inhibition of SP release is presumably involved in the suppression of PGE2, COX2, and inflammatory mediators, which are factors that enhance pain.

HSP 70 is expressed under various stress conditions, particularly induced by heat, ischemic, and oxidative stress, and is involved in the recovery response following cellular damage. Laser irradiation has been suggested to not only accelerate the healing process and reduce cellular damage by inducing stimulation of mitochondrial metabolism and an increase in electron transport but also promote the production of adenosine triphosphate (ATP) and growth factors, thereby activating efficient tissue repair [34]. It can be considered that the increase in ATP increases cellular respiration and suppresses hypoxia on the compression side during OTM. Therefore, OTM-induced ischemic stress and oxidative stress were alleviated by laser irradiation, and HSP70 expression was suppressed. Another study has suggested that HSP 70 reduces the activity of p38, c-Jun amino N-terminal kinase (JNK), and nuclear factor (NF)-κB signaling pathways [35]. Reduction in NF-κB signaling has been suggested to reduce the levels of TNF-α, IL-1β, matrix metalloproteinase-9, and other inflammatory mediators. Therefore, HSP 70 may be involved in reducing inflammatory mediators induced by ETM and reducing pain after the third day [18], but this needs to be evaluated over time. The results of the present study suggest that the expression of HSP 70 was reduced by laser irradiation because of the suppression of cell damage.

In this experiment, the temperature of the periodontal tissue of rats irradiated with the diode laser increased significantly after irradiation compared to that before irradiation (Figure 2). The maximum temperature of the periodontal tissue measured after irradiation was 34.0 °C. Although a study investigated the temperature changes caused by diode laser irradiation [36], none have reported significant temperature changes, and no study has demonstrated that temperature changes caused by diode laser irradiation may have affected pain. The study by Gunji et al. mentioned that blood flow increases when the tissue temperature is 40 °C or higher and does not change below 40 °C [37]. Therefore, although increased blood flow presumably reduces pain, the results of the present study suggest that the temperature changes caused by laser irradiation are unlikely to have affected the effectiveness of pain relief. Moreover, in recent years, high-frequency near-infrared (NIR) diode lasers capable of emitting ultra-short pulse waves have been developed, making it possible to penetrate light energy into tissue more efficiently without causing thermal damage. Therefore, pain relief achieved with NIR lasers is caused by the laser directly reaching the area of pain.

Nakatani et al. investigated the pain-relief benefits of NIR diode lasers [10]. The ETM model was similar to that used in the present study, and a laser with a wavelength of 910 nm (pulse duration 200 ns, 40 kHz, 120 J/cm^2^) was used. Tsuchiya et al. compared the effects of CO_2_ (wavelength 10,600 nm, 0.5 W, continuous wave, 30 or 600 s) and diode (wavelength 808 nm, 0.5 W, continuous wave, 30 or 600 s) laser irradiation on pain in rats undergoing ETM with 50 g, which is the same force employed in the present study [36]. According to these studies, both CO_2_ and diode lasers provide relief for pain induced by ETM. The pain relief of the diode laser device Sheep, which is a medical device similar to Sheep 810 used in this study, is set at an energy of 97.5 J/cm^2^ per irradiation. Since the Sheep 810 and Sheep have similar wavelengths, pulse widths, and frequencies, we set the irradiation conditions in the same way so that pain relief could be achieved without any safety issues. Few studies have examined the effect of diode laser irradiation on pain relief for ETM-induced pain. Further research is warranted into the appropriate irradiation time and power for pain relief using diode lasers.

The present study suggests that periodontally irradiated NIR diode laser irradiation may reduce pain induced by tooth movement through neuropeptide markers in the periodontal tissues. Photobiomodulation has the potential to improve outcomes as an adjuvant or alternative treatment for orthodontic treatment pain relief and may be efficacious as an application to improve the quality of treatment. However, there is insufficient evidence for the efficacy of photobiomodulation for orthodontic treatment. Therefore, clinical and basic studies elucidating the detailed mechanism of lasers in pain reduction are warranted to establish the optimal protocol.

This in vivo study has a few limitations and clinical implications. First, the study was determined to be minimally sampled, taking into account previous reports and 3R perspectives in animal studies. A post-hoc analysis of G-power showed that there may be a slight shortage of samples. Therefore, the results of this study should be interpreted with caution and taken as a slightly lower confidence value. Second, because this study aimed to evaluate the effect of laser irradiation on experimental tooth movement, the reaction to laser irradiation when no orthodontic force was applied to the teeth or periodontal tissues was not examined. However, investigating and comparing the changes observed when laser irradiation was applied to untreated periodontal tissues may lead to elucidation of the effects of laser irradiation on periodontal tissues. Therefore, it is considered worthwhile to investigate this in the future. Third, validation of animal studies to date suggests that periodontally irradiated laser irradiation may reduce pain when teeth move, via peripheral and central tissues, and the mechanism has been clarified. Future studies may need to investigate the optimal wavelength and irradiation conditions to reduce pain induced by tooth movement. Furthermore, with regard to pain reduction, further detailed mechanistic elucidation of the effects on peripheral inflammatory cytokines and neuropeptides in periodontal tissues and the responses of neurons at central projection sites is warranted. Finally, pain relief and laser irradiation conditions from animal studies may not be consistent with those in humans. Therefore, it is necessary to pay close attention to the application of the results obtained in animals to humans. Low-power laser therapy is a highly safe treatment with less invasion and side effects on living organisms, but each laser has a specific wavelength and characteristics. Tissue penetration and surface resorption also have different effects on different tissues. A possibility of burning or damage to the tissue exists, especially when the energy of the laser increases. Therefore, it is necessary to understand the characteristics of the laser as well as consider the amount of energy. Moreover, further clinical research, which is blinded in accordance with the law, is warranted for clinical application in humans.

In the future, further high-quality clinical studies, along with the elucidation of the detailed effect of lasers on pain through basic research, will aid in establishing the optimal protocol.

## 4. Materials and Methods

### 4.1. Animals

We obtained 7-week-old male Sprague–Dawley rats (10 rats in each group, 20 rats in total) (Japan SLC, Shizuoka, Japan). After preliminary breeding for 1 week, 8-week-old rats were used in this study. The rats were reared at the Kasumi Animal Experiment Facility, Life Science Research Section, Natural Science and Research Support Center, Hiroshima University, Japan. The study protocol was approved by the Ethics Committee for Animal Experiments of the Hiroshima University School of Dentistry (approval number: A23-122).

### 4.2. ETM Model of Rats

**Figure 6:**
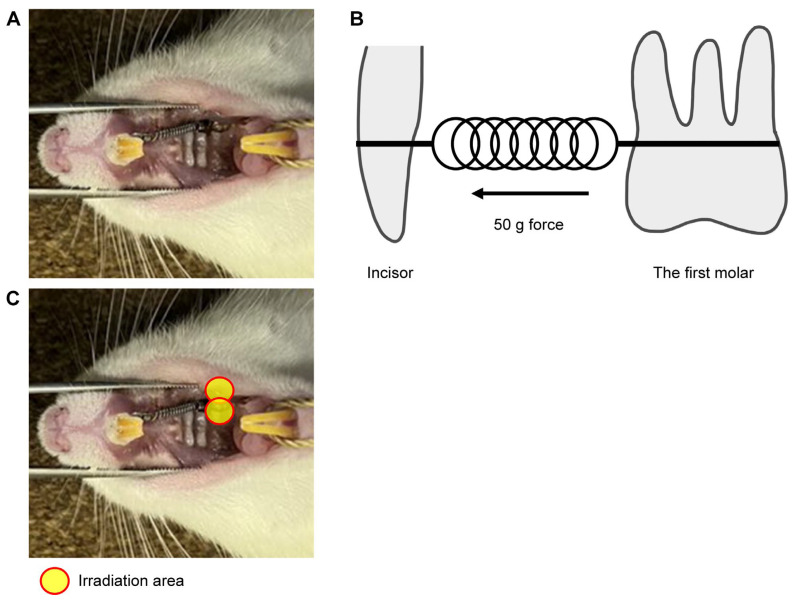
Experimental tooth model. (**A**) A 50-gF nickel–titanium (Ni-Ti) closed-coil spring was applied between the upper left first molar and upper incisors of the rat using an orthodontic ligature wire. (**B**) The buccal and palatal sides of the first molar were irradiated for 45 s in each area, and the total energy was set to 100.8 J/cm^2^. (**C**) In vivo model of experimental tooth movement. A Ni-Ti closed-coil spring was used, and the orthodontic force was set to 50 g. The ligatures were bonded with resin cement to prevent detachment of the appliance.

A nickel–titanium (Ni-Ti) closed-coil spring (inner diameter: 0.9 mm, wire diameter: 0.23 mm, Tomy International, Tokyo, Japan) was ligated between the upper left first molar and upper incisors of the rat using an orthodontic ligature wire (Tomy International) as shown in Figure 6A. The ligatures were bonded with resin cement (Superbond; Sun Medical, Moriyama, Japan) to prevent detachment of the appliance. The orthodontic force was set to 50 g (Figure 6B). The rats were anesthetized via isoflurane inhalation and intraperitoneal administration of three anesthetics (midazolam, medetomidine, and butorphanol). The rats in the ETM + laser group were irradiated with the diode laser following ETM. From the viewpoint of the ‘3R’ of experimental animals, the right side without ETM treatment was considered the untreated control group.

### 4.3. Laser Irradiation

A gallium–aluminum–arsenide (Ga-Al-As) diode laser (Sheep 810; UNITAC, Hiroshima, Japan) with a wavelength of 808 nm, peak power of 7 W, average power of 560 mW, and pulse duration of 20 ms was employed. Laser irradiation was performed using a probe. The buccal and palatal sides of the first molar were irradiated for 45 s for each area (Figure 6C) with 1.12 mW/cm^2^ power density and 100.8 J/cm^2^ total energy density for a total of 90 s. Laser irradiation was performed immediately after ETM, and contact of the irradiation port with the gingiva was constantly maintained during irradiation. Table 1 summarizes the specifications of the physical parameters of the laser, energy density, and doses per point of irradiation.

### 4.4. Behavior Testing

As signs of nociception, the number of facial groomings and VCM periods was measured for 10 min in a transparent plastic cage (25.0 cm × 19.8 cm × 19.2 cm). Prior to the experiment, all of the rats were allowed to acclimatize for 10 min. Measurements of nociceptive behavior were performed before the ETM (T0) and 24 h after fixation of the appliance (T1).

### 4.5. Temperature Measurement

The temperature around the upper left first molar of the rat, which was the site of laser irradiation, was measured by using the thermography camera FLIR C5 (FLIR Systems, OÜ, Estonia). The distance between the first molar and the camera was 14 cm. The measured temperature is displayed in the upper left corner of the camera (Figure 2B,C). The measurements were performed before and immediately after the laser procedure.

### 4.6. Examination of Histological Changes

The rats were anesthetized and perfused with saline, followed by perfusion fixation in 4% paraformaldehyde, 0.1 M phosphate buffer (pH 7.4), 24 h following ETM. The maxillary bones were removed and fixed in 4% neutral buffered paraformaldehyde solution (Wako Pure Chemicals Co., Osaka, Japan) for 24 h at 4 °C and then decalcified in 14% tetrasodium ethylene diamine tetra acetic acid solution (Sigma-Aldrich, St Louis, MO, USA) for 8 weeks at 4 °C. Subsequently, paraffin embedding was performed using an automatic embedding machine Excelsior AS (Thermo Fisher Scientific, Waltham, MA, USA), and 7-μm-thick horizontal continuous sections were prepared using a rotary microtome (Microm HM 325; Carl Zeiss, Oberkochen, Germany). In the ETM model employed in this study, the inclination of the first molar was induced by traction force. The center of rotation was the point one-third from the root apex of the mesial root. Therefore, compression was generated mesially and traction was generated distally on the coronal side. Traction was generated mesially and compression was generated distally on the apical side. In this study, coronal sections were prepared, and the histological changes were evaluated according to the methods described by Kawasaki et al. and Gunji et al. [37,38].

### 4.7. Immunohistochemical Staining

After deparaffinization with xylene, the slides were hydrophilized using ethanol. To inhibit endogenous peroxidase activity, treatment with 80% methanol containing 0.3% hydrogen peroxide (Sigma-Aldrich) was performed for 15 min, followed by washing and incubating with 5% bovine serum albumin (BSA) for 10 min to prevent non-specific reactions. Anti-SP (1:500; ab14184, Abcam, Cambridge, UK), anti-CGRP (1:1000; ab47027, Abcam), and anti-HSP 70 (1:200; ab2787, Abcam) antibodies were incubated at 4 °C for 24 h. The primary antibodies were diluted in 5% BSA. After washing with sterile phosphate-buffered saline (PBS), each section was incubated with secondary antibodies, anti-mouse IgG Histofine Simple Stain MAX-PO (M) (Nichirei Bioscience, Tokyo, Japan), and anti-rabbit IgG Histofine Simple Stain MAX-PO (R) (Nichirei Bioscience) for 30 min at room temperature and then washed with PBS. A positive reaction to the primary antibody was confirmed by coloration using a Histofine DAB Substrate Kit (Nichirei Bioscience). After counterstaining with hematoxylin staining solution (Sakura Finetek Japan Co., Tokyo, Japan) and dehydration with ethanol, the sections were mounted on coverslips using Entellan New (Sigma-Aldrich). The tissue sections were observed under an all-in-one microscope BZ-X800 (Keyence, Osaka, Japan). The number of positive cells in the periodontal ligament on the compressed side was counted. The average of five sections was used as the representative value for each rat, and statistical analysis was performed for five rats in each group.

### 4.8. Statistical Analysis

Data are expressed as mean ± standard deviation. The Wilcoxon signed-rank test was used for comparisons between T0 and T1 within groups. Statistical analyses were performed using two-factor analysis of variance (ANOVA). If any ANOVA tests demonstrated significant results, pairwise multiple comparisons (Tukey’s test) were performed to identify significantly different variables. Significance levels were set at *p* < 0.05 or *p* < 0.01.

## 5. Conclusions

The present study has demonstrated that diode laser irradiation of periodontal tissues decreased SP, CGRP, and HSP 70 expression in the ETM rat model, indicating that the diode laser could reduce pain and suppress cell damage in the periodontal tissue during orthodontic treatment.

## Figures and Tables

**Table 1:** Description and specification of the physical parameters of the laser, energy density, and dose per point of irradiation.

Parameter	Value
Wavelength	808 nm
Operation mode	Pulse
Pulse duration	20 ms
Peak power	7 W
Average power	560 mW
Frequency	5 Hz
Power density	1.12 W/cm^2^
Irradiation diameter	0.8 cm
Beam area	0.5024 cm^2^
Irradiation time	45 s
Energy density	50.4 J/cm^2^

## Data Availability

The data presented in this study are available on request from the corresponding author.

## Abbreviations

The following abbreviations are used in this manuscript:

HSP	heat shock protein
ETM	experimental tooth movement
VCM	vacuous chewing movement
IL	interleukin
SP	substance P
ASIC3	acid-sensing ion channel 3
TRPV1	transient receptor potential vanilloid 1
CGRP	calcitonin gene-related peptide
TG	trigeminal ganglion
SGCs	satellite glial cells
TNFα	tumor necrosis factor-α
MAPK	mitogen-activated protein kinase
NIR	frequency near-infrared
